# COVID-19 Stigma Induced by Local Government and Media Reporting in Japan: It’s Time to Reconsider Risk Communication Lessons From the Fukushima Daiichi Nuclear Disaster

**DOI:** 10.2188/jea.JE20200247

**Published:** 2020-08-05

**Authors:** Takashi Yoshioka, Yohei Maeda

**Affiliations:** 1 Center for Innovative Research for Communities and Clinical Excellence (CiRC^2^LE), Fukushima Medical University, Fukushima, Japan; 2 Department of Otorhinolaryngology-Head and Neck Surgery, Osaka University Graduate School of Medicine, Osaka, Japan

As the COVID-19 pandemic continues to evolve globally and fear of infection increases, the stigmatization of people, driven by fear, has become a serious problem in some societies.^1^ In Japan, a specific COVID-19 stigma problem has arisen: stigma induced by local government and media reporting.

COVID-19 stigma was first reported at the end of March, 2020. University staff of Koriyama Women’s University in Fukushima Prefecture and students at an affiliated high school were ridiculed following news that a professor at the school was diagnosed with the disease.^2^ Another case was reported from Kyoto Sangyo University in Kyoto Prefecture. After news that a COVID-19 cluster originated from some students, students at the university were defamed directly, or by social media, and the university received threating messages from unknown individuals.^3^

These reports share two common features. First, the local governments revealed detailed information about the patients, including their age, sex, workplace, and school, in addition to their behavioral histories. Second, this information was immediately spread via mass media and social media. Numerous people saw this information, giving them access to the patients’ detailed personal information, which resulted in stigma and discrimination.

Some groups of Japanese people, especially in Fukushima, experienced a similar stigma after the Fukushima Daiichi Nuclear Disaster in 2011. Many people around the world were influenced by the numerous—and sometimes unsubstantiated—details reported in the mass media and social media. As a result, many Fukushima residents experienced groundless stigmatization, particularly with regard to the effects of radiation.^4^ Such stigma psychologically impacted the residents of Fukushima, resulting in many people requiring mental health care or treatment.^4^

One way to reduce such stigma is to limit reporting of personal information while promoting accurate social risk communication. In line with a World Health Organization’s statement,^5^ individuals should not share unconfirmed rumors, and should instead refer to scientifically based topics related to COVID-19, both in real life and on social media. Meanwhile, to prevent social stigma associated with COVID-19, local governments and mass media should be mindful of journalism ethics—for example, avoiding focusing on individuals’ behaviors or patients’ responsibility for becoming infected with COVID-19. It is also important to protect people from the stigma that has already arisen. According to the Centers for Disease Control and Prevention in the United States,^6^ coping with stress associated with COVID-19 and speaking out against stigmatizing behaviors, even on social media, are vital.

Eliminating stigma is one of the most important global issues associated with COVID-19. Stigmatization must stop, as it only adds stress and suffering to this difficult situation.^7^ To counter the stigma that has unfortunately arisen, we must cope with the stress individually and strengthen the community socially, as shown in Table 1.^5^^,^^6^ Now is the time to reconsider how we manage risk communication.

**Table 1.** Recommendations for preventing and addressing stigma associated with COVID-19

No.	Recommendation
1	Ensure the privacy and confidentiality of individuals.
2	Avoid using certain words which may have a negative meaning and drive stigmatizing attitudes.
3	Promote and spread accurate information about prevention, lifesaving actions, screening, and treatment.
4	Correct misconception, rumors, and misinformation that can cause stigma.
5	Speak out against negative behaviors both in real life and on social media.
6	Make sure to portray diverse ethnic communities when using images.
7	Share voices and stories that humanize the experiences and struggles of people or groups affected by COVID-19.
8	Communicate support and encouragement for healthcare workers, responders, and others working on the front lines.
9	Suggest resources for mental health or other social support services for those who have experienced stigma.
